# Exercise-Related Changes of Networks in Aging and Mild Cognitive Impairment Brain

**DOI:** 10.3389/fnagi.2016.00047

**Published:** 2016-03-07

**Authors:** Pei Huang, Rong Fang, Bin-Yin Li, Sheng-Di Chen

**Affiliations:** ^1^Department of Neurology and Institute of Neurology, Ruijin Hospital, Shanghai Jiao Tong University School of MedicineShanghai, China; ^2^Department of Neurology, Ruijin Hospital and Ruijin Hospital North, Shanghai Jiao Tong University School of MedicineShanghai, China

**Keywords:** exercise, aging, mild cognitive impairment, functional magnetic resonance imaging, electroencephalogram, magnetoencephalography, positron emission tomography

## Abstract

Aging and mild cognitive impairment (MCI) are accompanied by decline of cognitive functions. Meanwhile, the most common form of dementia is Alzheimer’s disease (AD), which is characterized by loss of memory and other intellectual abilities serious to make difficulties for patients in their daily life. MCI is a transition period between normal aging and dementia, which has been used for early detection of emerging dementia. It converts to dementia with an annual rate of 5–15% as compared to normal aging with 1% rate. Small decreases in the conversion rate of MCI to AD might significantly reduce the prevalence of dementia. Thus, it is important to intervene at the preclinical stage. Since there are still no effective drugs to treat AD, non-drug intervention is crucial for the prevention and treatment of cognitive decline in aging and MCI populations. Previous studies have found some cognitive brain networks disrupted in aging and MCI population, and physical exercise (PE) could effectively remediate the function of these brain networks. Understanding the exercise-related mechanisms is crucial to design efficient and effective PE programs for treatment/intervention of cognitive decline. In this review, we provide an overview of the neuroimaging studies on physical training in normal aging and MCI to identify the potential mechanisms underlying current physical training procedures. Studies of functional magnetic resonance imaging, electroencephalography, magnetoencephalography and positron emission tomography on brain networks were all included. Based on our review, the default mode network, fronto-parietal network and fronto-executive network are probably the three most valuable targets for efficiency evaluation of interventions.

## Introduction

Aging is characterized by a progressive decline of physiological efficiency in cells and tissues that increases the risk of disease and death, which is not pathological but obligatorily normal (Harman, 2001). Decline of cognitive functions, along with structural and functional changes in brain regions, are accompanying symptoms of aging in humans (Hedden and Gabrieli, 2004). Age-related cognitive decline is mainly associated with working memory, executive function and episodic memory (Schaie, 1994). Meanwhile, the most common form of dementia is AD, which is characterized by loss of memory and other intellectual abilities serious to make difficulties for patients in their daily life. AD is not only a cognitive problem, but also a main cause of mortalities in older adults. Until 2013, the number of AD patients has reached 35 million worldwide, and this number is supposed to triple by 2050 (Hosseini et al., 2014; James et al., 2014). However, there are still no effective disease-modifying drugs for AD.

Mild cognitive impairment represents a transitional condition between normal aging and mild dementia, which has been used for early detection of emerging dementia (Petersen et al., 2001, 2009; Guillozet et al., 2003). Indeed, MCI patients already show significant lesion loads (Jack et al., 2013; Villemagne et al., 2013). The most common form of MCI is amnestic mild cognitive impairment (aMCI), which is characterized by slight memory impairment with other cognitive domains being relatively preserved (Petersen et al., 2009; Dubois et al., 2010). It converts to dementia with an annual rate of 5–15% as compared to normal aging with 1% rate (Petersen et al., 2009). Previous studies demonstrated that small decreases in the conversion rate of MCI to dementia might significantly reduce the prevalence of dementia (Ferri et al., 2005). Thus, MCI has been recognized as a target for interventions to slow down the progression of cognitive decline to dementia (Petersen and Morris, 2005).

Since the number of dementia is rapidly growing and there are still no effective drugs to treat it, it is necessary to intervene at the preclinical stage. Non-drug intervention is important for the prevention and treatment of cognitive decline of aging and MCI. Many studies suggest that PE, specifically incorporating aerobic exercise, might lead to cognitive improvement in normal aging, MCI and AD (Kramer and Erickson, 2007; Hillman et al., 2008; Lautenschlager et al., 2008; van Uffelen et al., 2008; Baker et al., 2010; Suzuki et al., 2012; Bherer et al., 2013). Exercise can contribute to enhanced blood flow and changes in the brain environment, leading to the restoration of physiological and structural function (Petzinger et al., 2013). Functional MRI data showed that neuronal activity in the prefrontal regions of normal aging and AD became more efficient after aerobic exercise (Colcombe et al., 2004; Angevaren et al., 2008). Aerobic exercise might restore cognition of normal aging and AD through the promotion of neuroplasticity (Turner and Spreng, 2012). Increasing evidences demonstrated exercise-related improvements in cognitive performance and revealed exercise-related changes in neuroimaging biomarkers in normal aging, MCI and early stages of AD (Kramer and Erickson, 2007; Hillman et al., 2008; Lautenschlager et al., 2008; van Uffelen et al., 2008; Baker et al., 2010; Suzuki et al., 2012; Bherer et al., 2013). It is important to investigate neural networks that are affected in normal aging and MCI, and those rehabilitated by the training procedure. Understanding the exercise-related mechanisms is crucial to design efficient and effective PE programs for treatment/intervention of cognitive decline.

In this review article, we provide an overview of the neuroimaging studies on physical training in normal aging and MCI to identify the potential mechanisms underlying current physical training procedures. We will then discuss the common findings of the current studies and address the implications for future research.

## Aging and Mild Cognitive Impairment-Related Biochemical Changes

The major pathological features in AD brains are the presence of intra-neurofibrillary tangles and extracellular protein Aβ deposits, especially in the regions related to learning, memory, and cognitive functions (Reddy and McWeeney, 2006). The distribution of amyloid deposits in MCI was reported to be intermediate between the changes in normal aging and AD brain (Markesbery, 2010). Growing evidences suggested that aging was a key factor for the increase in production of Aβ and the decreased Aβ-degrading enzymes in the AD brain (Reddy et al., 2010). Studies of postmortem brains from aged humans with MCI/AD found that Aβ levels increased with age (Gouras et al., 2005). In addition, AD patients differed from normal controls in CSF proteins, with early increase of Aβ42 and later decrease of t-tau and p-tau (Antonell et al., 2011). Those CSF biomarkers also showed good diagnostic accuracy and prediction of conversion from MCI to AD (van Harten et al., 2013; Dubois et al., 2014).

## Aging and Mild Cognitive Impairment-Related Changes of Brain Networks

The field of systematic neuroscience has been revolutionized by functional neuroimaging in recent years. Techniques such as fMRI, EEG, MEG, and PET now provide researchers with unprecedented information on the mechanisms of human brain function. In the following, we provide an overview of neuroimaging studies, which observed brain network changes in normal aging and MCI, to understand the underlying mechanisms of cognitive decline (**Table 1**).

**Table 1:** List of included studies on aging and MCI-related network changes.

Neuroimaging techniques	Reference	Target	Control	Cognitive task	Mainly related brain regions/networks
fMRI	Cabeza et al., 2002	Low-performing and high-performing older adults	Young adults	Recall and source memory	Prefrontal cortex (PFC)
fMRI	Madden et al., 2010	Older adults	Young adults	Task switching	Fronto-parietal (FP) network
fMRI	Grady et al., 2010	Aging	Younger adults	Visual tasks: detection, perceptual matching, attentional cueing, and working memory	The DMN and the task-positive network (TPN)
fMRI	Bai et al., 2011	Amnestic mild cognitive impairment (aMCI)	Normal aging	None	Temporal correlations, subcortical and frontal cortex
fMRI	Turner and Spreng, 2012	Older adults	Young adults	Executive control processing (including working memory and inhibition)	Dorsolateral prefrontal cortex, supplementary motor cortex and left inferior parietal lobule (working memory); right inferior frontal gyrus and presupplementary motor area (inhibition)
fMRI	Onoda et al., 2012	Aging	Younger adults	Resting-state	Salience network (consisted of the bilateral insula and the anterior cingulated cortex), internetwork connectivities (salience to auditory, default mode to visual, etc.)
fMRI	Tomasi and Volkow, 2012	Aging	Younger adults	Resting-state	DMN, dorsal attention network (DAN), somatosensory and subcortical networks
fMRI	Zhou et al., 2013	MCI and AD	Normal cognition	Resting-state	Thalamo-default mode network and thalamocortical connectivity
fMRI	Liu et al., 2014a	AD and aMCI	Normal aging	Resting-state	DMN, global and nodal network efficiency
fMRI	Zhou et al., 2015	AD and MCI	Normal cognition	Resting-state	Functional connectivities throughout the brain (included DMN, temporal lobe)
EEG	Kikuchi et al., 2000	Aging	Younger adults	At rest and during photic stimulation	Interhemispheric functional connectivity
EEG	Gaál et al., 2010	Elderly subjects	Younger subjects	While eyes opening	Posterior area, frontal area
EEG	Deiber et al., 2013	Elderly adults	Young adults	The attention network test (ANT)	Midparietal alpha inhibition and posterior alpha activation
EEG	Gola et al., 2013	Elderly subjects	Young controls	Simple visual attention task	Beta-band power of EEG signals recorded over occipital regions
EEG	Vecchio et al., 2014	AD and MCI	Elderly subjects	–	Delta, theta, and alpha 1 bands of brain cortical recordings
EEG	Knyazev et al., 2015	Elderly subjects	Young controls	–	Beta and gamma band networks (including posterior cortical regions and anterior attentional system)
EEG	Moretti, 2015	MCI due to AD	–	–	Temporoparietal area
MEG	Buldú et al., 2011	MCI	Healthy controls	During a memory task	The whole functional network architecture
MEG	López et al., 2014	Aging with high cognitive reserve (CR)	Aging with low CR	–	The dynamics of functional connectivity
MEG	Pineda-Pardo et al., 2014	aMCI	Healthy controls	Resting-state	The whole brain connectivity
PET	Seo et al., 2013	MCI and AD	Cognitively normal	Resting-state	A small-world property, the DMN
PET	Oh and Jagust, 2013	Normal older people	Young subjects	Memory encoding of visual scenes	Task-independent functional connectivity between parahippocampal gyrus and prefrontal cortex
PET	Sanabria-Diaz et al., 2013	MCI and AD	Normal healthy controls	Resting-state	Global and local network properties
PET	Liu et al., 2014b	Normal aging	Younger subjects	Resting-state	A small-world network, association and paralimbic cortex regions

### Functional Magnetic Resonance Imaging

Previous studies have shown that episodic memory, processing speed, executive function, and other cognitions declined with aging (Hedden and Gabrieli, 2004; Nyberg et al., 2012). FMRI including resting-state or task-based serials is widely used to investigate the relationships between brain networks and cognition. Tambini et al. (2010) performed resting-state fMRI scans in healthy persons, and found enhanced functional connectivity between hippocampus and the lateral occipital complex associated with higher subsequent memory scores after associative encoding tasks. Sala-Llonch et al. (2015) proved that the strength of connectivity between hippocampus and other cortex was weaker with aging (Madden et al., 2010). At the meantime, some studies found that elderly persons had higher activities in some brain domains using task-based fMRI, which reflected a compensatory mechanism when the functional connectivity of brain was disrupted (Grady, 2012). The findings above might explain the mechanisms of poor cognitive functions in aging. But the changes of brain activities in normal aging were very complex. Grady (2012) found that increased brain activity in elderly adults could be associated with either better or worse task performance. The truth is that aging could be influenced by a large number of factors, including education, life experiences, diet, and genes. Nevertheless, many studies have reached an agreement that normal aging population showed altered patterns of brain activity compared to young adults when performing cognitive tasks (Turner and Spreng, 2012). The phenomenon of different patterns of brain activity in aging population could be explained as follows: (1) the differentiation hypothesis: it has been suggested that a decline in dopaminergic neuromodulator could lead to less neural noise, which directly showed less distinctive cortical activities associated with deficits in cognition (Li et al., 2001); (2) the compensation hypothesis: the elderly adults recruited more strength of network than younger adults but inefficiently, especially in prefrontal cortex (Cabeza et al., 2002). In general, task-based fMRI reflects the activities of different brain regions while resting-state fMRI calculates the connectivity magnitude between brain regions. Different models of fMRI can investigate different aspects of the brain networks and we should use them combined.

Mild cognitive impairment is considered as a transition period between normal aging and dementia, especially, the aMCI has a high risk of progression to AD (Petersen et al., 2009). So studies focusing on the brain networks of MCI help us understand the pathological cascading of AD. Some fMRI studies have reported that the strength of functional connectivity, which was positively correlated with the scores of the mini-mental state examination (MMSE), was weaker in MCI than normal aging (Bai et al., 2011; Zhou et al., 2013, 2015). Bai et al. (2011) revealed abnormal interregional correlations in the whole brain areas of MCI patients when compared to normal aging populations, particularly in subcortical regions and frontal cortex. In addition, they also found that negative functional connections decreased with the progression of disease (Bai et al., 2011). There may be a compensatory mechanism during the process of MCI as in normal aging. Besides, Liu et al. (2014a) proved that the loss of long-distance connectivity was related to the severity of MCI and AD (Liu et al., 2014a).

The most commonly studied brain network using resting-state fMRI is the DMN, which has the advantage in reflecting internal cognition at rest. DMN is the most relevant network model reflecting the functional and pathological cascade underlying AD (Buckner et al., 2009). Buckner et al. (2008) found that the DMN mainly included the medial temporal lobe and the medial prefrontal subsystems. There was evidence that the DMN was overlapped with brain areas that had the deposition of CSF biomarkers, including Aβ42, t-tau and p-tau (Li et al., 2014). Functional connectivities within the DMN were found reduced in older adults compared with the younger adults (Grady et al., 2010). This helps us further understand MCI and AD, which are both described as disconnective syndromes (Seeley et al., 2009). Besides DMN, other intrinsic brain networks have also been studied in aging. Yeo et al. (2011) organized the cortex into seven networks coarsely and 17 networks at a finer level according to 1,000 individuals. Onoda et al. (2012) reported that salience network and other internetworks were also disrupted in elders, which were related to cognitive decline. Tomasi reported that aging had severer impact on the long-range functional connectivity density (FCD) than on the short-range FCD, indicating that long-range networks might be more vulnerable to aging (Tomasi and Volkow, 2012). Similarly, anterior and posterior components of the DMN, which are long-distance connectivities, are destructed in MCI patients. And severer reduction of global and nodal network efficiency was found in MCI and AD compared to normal aging (Liu et al., 2014a). Consistent with previous results, the connectivities of several important nodes within the DMN in MCI and AD patients had been found inefficient by Zhou et al. (2015) such as the posterior cingulate gyrus, the precuneus, the parahippocampal gyrus, and the medial superior frontal gyrus. These findings support that human brain is the integration of neural networks and MCI is a prodromal stage of AD (Gauthier et al., 2006).

### Electroencephalogram

EEG is a useful tool in detecting cognitive level and brain networks (Gaál et al., 2010). van der Hiele et al. (2008) reported that baseline EEG,which showed increased theta activity (4–8 Hz) during eyes closed and less alpha reactivity (8–13 Hz) during eyes open and memory activation, could be markers of future cognitive level of the elderly. Kikuchi et al. (2000) found that there was a lower coherence in the resting EEG for the delta, theta, alpha-3, beta-1 and beta-2 frequency bands, which reflected a weaker interhemispheric functional connectivity in elderly subjects. Gaál et al. (2010) pointed out decreased values of the clustering coefficient, path length and the “small-world index” when elders were opening eyes compared with younger subjects. It also suggested a reduced reactivity with aging and a decreased level of integrative activity of the brain (Gaál et al., 2010). Besides, an overall reduction of task-related alpha activity in brain circuits was found in the elderly compared with the youngers during attentional functions (Deiber et al., 2013). The beta-band activity of EEG in elderly subjects was also found to be decreased, which proved a deficit of aging population in attention processes (Gola et al., 2013). Furthermore, Knyazev et al. (2015) found a decrease of modularity and clustering in beta and gamma band networks in aging using Graph-theoretical analysis with EEG, which implied that aging made brain networks more randomly.

EEG has also been used to detect the brain cognitive networks of MCI patients. Vecchio et al. (2014) discovered that MCI subjects were significantly impaired in theta but not alpha bands connectivity compared with normal aging with graph theoretical analysis. A study of EEG revealed that upper/low alpha power ratio could predict MCI, which was associated with cortical thinning and less perfusion in the temporoparietal area (Moretti, 2015).

### Magnetoencephalography

MEG is another important tool to study the brain networks and cognitive functions of aging and MCI, which records the neuronal activity reflecting the dynamics of the cortical networks (López et al., 2014). A study of the neural activities using MEG indicated that alpha oscillatory activity in DLPFC was synchronized with that in parietal regions during visual short-term memory (Grimault et al., 2009). Besides, it was verified that parieto-occipital alpha power was markedly stronger for successfully encoded long-term memory sequences (Meeuwissen et al., 2011). A research of aging populations revealed that the subjects with lower cognitive reserve exhibited higher functional connectivity than those with higher cognitive reserve in MEG signals (López et al., 2014). This can be explained by a way of compensation.

In the MEG study of brain networks in MCI, Buldú et al. (2011) reported that MCI patients showed an enhancement of the strength of connections, together with an increase in the outreach parameter. In addition, scientists proposed a structural connectivity of the graphical lasso (GL), in which region-specific time series were obtained in five different frequency bands. The results concluded that the structural connectivity could estimate functional activity, and this classification method of MCI was better than traditional ways (Pineda-Pardo et al., 2014).

### Positron Emission Tomography

Positron emission tomography is also an important tool to investigate the brain networks, which reflects the cerebral glucose metabolism sensitively (Liu et al., 2014b). Previous study certified that the index of blood-flow tested by PET was consistent with brain anatomical connectivity (Ferrarelli et al., 2004). Liu et al. (2014b) reported that both of younger adults and elderly subjects showed small-world architecture in brain networks utilizing PET, but increased clustering and decreased efficiency were found in elderly subjects. Taking advantage of Pittsburg compound B-positron emission tomography (PiB-PET), Oh and Jagust (2013) certified that elderly adults without Aβ deposition showed decreased regional brain activation and decreased functional connectivity between parahippocampal gyrus and prefrontal cortex compared with young subjects.

PET with ^18^F-fluorodeoxyglucose ([^18^F]FDG-PET), which is a marker of synaptic function, measures regional brain glucose metabolism (rBGM). Hypometabolism on [^18^F]FDG-PET is related to neuronal dysfunction and neurodegeneration (Herholz et al., 2011; Jack et al., 2013). A reduction in rBGM in the posterior cingulate and temporoparietal regions was related to a faster cognitive decline in MCI patients (Minoshima et al., 1997; Drzezga et al., 2003). Seo et al. (2013) reported that MCI and AD had the same ‘small-world’ property of whole-brain network as cognitively normal populations using [^18^F]FDG-PET. However, local clustering of networks was lower and normalized betweenness centrality in hubs of DMN was decreasing in MCI compared to cognitively normal populations (Seo et al., 2013). Besides, Sanabria-Diaz et al. (2013) detected the global and local network properties (global and local efficiency, clustering index, and others) in MCI using [^18^F]FDG-PET, and certified that the network of MCI was in the transitional stage between cognitively normal populations and AD.

## Exercise-Related Changes of Networks in Aging and Mild Cognitive Impairment

Exercise has been found to lead to cognitive improvement in normal aging, MCI and AD with increasing evidences demonstrated exercise-related changes in imaging biomarkers. In order to understand the exercise-related mechanisms underlying cognitive improvements, we provide an overview of neuroimaging studies focusing on brain network changes caused by exercise in normal aging and MCI (**Table 2**).

**Table 2:** List of included studies on exercise-related changes of networks in aging and MCI.

Neuroimaging techniques	Reference	Target	Control	Exercise	Duration	Cognitive task	Mainly related brain regions/networks
fMRI	Voss et al., 2010b	Aerobic walking older adults	Non-aerobic stretching older adults	Walking	1 year	Digit span task, Task switching, Wisconsin Card Sorting Task (WCST), Spatial memory	Default mode network, frontal executive (FE) network, frontal parietal (FP) network
fMRI	Voss et al., 2010a	Healthy elderly adults	–	Aerobic fitness	–	Task switching, Wisconsin Card Sorting Task (WCST), Spatial memory	Default mode network
fMRI	Taubert et al., 2011	Young adults-motor training	Yong adults-no training	Dynamic balancing task	6 weeks	Acquired motor skill	Increased fronto-parietal network connectivity
fMRI	Voss et al., 2011	Higher-fit children	Lower-fit children	Aerobic fitness	–	Cognitive control task	Dorsal anterior cingulate, putamen, central opercular
fMRI	Zlatar et al., 2013	Physically active older adults	Sedentary older adults	–	–	Semantic fluency task	Attention and language networks
fMRI	Wei et al., 2013	Tai Chi Chuan (TCC) practitioners	TCC-naïve Controls	Long term TCC	–	Attention network test	Thicker cortex in left medial occipito-temporal sulcus and lingual sulcus
fMRI	Wei et al., 2014	Aging TCC practitioners	Aging TCC-naïve controls	Long term TCC	–	Attention network test	Increased functional homogeneity in the post-central gyrus (PosCG)
fMRI	Smith et al., 2013	MCI-exercise	Normal control-exercise	Treadmill walking	12 weeks	Semantic memory task	Frontal, temporal and parietal lobes
fMRI	Suzuki et al., 2013	MCI-exercise	MCI-naive	Multicomponent exercise	6 months	MMSE, ADAS-Cog, logical memory	Medial temporal areas including entorhinal cortex
EEG	Fong et al., 2014	Physically active older adults	Sedentary older adults	Endurance exercise, TCC	Long term exercise	Task-switching task	Frontal, central and parietal midline sites
EEG	Chang et al., 2015	Young adults with antecedent exercise	Young adults without antecedent exercise	Acute cycling exercise	–	Attention network test	Three attentional networks: alerting, orienting, and executive control
EEG	Luque-Casado et al., 2015	High-fit young adults	Low-fit young adults	Aerobic fitness	–	The Psychomotor Vigilance Task	Attentional networks
EEG	Smallwood et al., 2015	High-activity young adults	Low-activity young adults	Aerobic fitness	–	Visual-evoked potentials (VEPs)	Visual sensory long-term potentiation (LTP)
EEG	Bullock et al., 2015	High-intensity exercise	Low-intensity exercise	Acute bouts of aerobic physical exercise	–	Perceptual and cognitive processes	Parietal electrodes
EEG	Gajewski and Falkenstein, 2015	Active aging	Inactive aging	Lifelong habitual physical activity	–	Memory-based task switching	Frontal electrodes
EEG	Hogan et al., 2015	High-fit adolescent	Low-fit adolescent	Aerobic fitness	–	An executive function task	Frontal area
MEG	Douw et al., 2014	Healthy persons	–	Physical fitness	–	Dutch intelligence test	Increased intermodular connectivity in the beta band
PET	Schultz et al., 2015	High- cardiorespiratory fitness aging at risk for AD	Low- cardiorespiratory fitness aging at risk for AD	A graded treadmill exercise (VO2peak)	–	A comprehensive neuropsychological exam (immediate memory, verbal learning and memory)	Increased PiB-PET binding and reduced CSF Aβ42
PET	Porto et al., 2015	Pre-training MCI	After-training MCI	Aerobic training	24 weeks	MMSE, ADAS-Cog	Dorsal anterior cingulate cortex

### Functional Magnetic Resonance Imaging

Functional MRI data have demonstrated exercise-related brain network changes in cognitive regions of healthy persons. Higher connectivity within the DMN has been associated with increased cardiorespiratory fitness, and DMN connectivity also mediates the relationship between maximal oxygen consumption (VO_2_ max) and cognitive functions (Voss et al., 2010a). Another study, which examined the effect of aerobic fitness on cognitive control in preadolescent children, found that high-fit children outperformed low-fit children on cognitive control, and individual differences in cognitive control performance were associated with aerobic fitness (Voss et al., 2011). Longitudinal functional and structural MRI study of young adults who accepted a 6-week motor training showed increased FPN connectivity in accordance with cognitive performance improvements. The structural gray matter alterations were also tightly correlated with functional connectivity changes in prefrontal and supplementary-motor areas (Taubert et al., 2011).

Studies on older adults also found that age-related dysfunction of brain networks were remediated by PE. Zlatar et al. (2013) found that during a semantic fluency task, inactive aging displayed reductions in negative task-related activity compared to the active aging in areas of the attention network. It indicated that exercise might remediate these alterations in network activity related to attention and language processing, physical activity may alleviate the impact of aging on language functions (Zlatar et al., 2013). Wei et al. (2014) found that Tai Chi Chuan (TCC) could influence the functional plasticity of the brain’s intrinsic architecture and optimize locally functional organization to improve cognition in aging population. Moreover, TCC also potentially increased the thickness of brain regions associated with motor and executive functions (Wei et al., 2013). After a 1-year exercise intervention in aging adults, both the DMN and the FPN exhibited higher connectivity than controls (Voss et al., 2010b).

Furthermore, some studies revealed changes in imaging biomarkers in subjects with MCI who performed PE. One study used structural MRI to reveal a lower rate of brain atrophy in the MCI population (Suzuki et al., 2013). Another study used functional MRI to show decreases in hippocampal activation during a semantic memory retrieval task, indicating that neural efficiency was improved after the PE intervention (Smith et al., 2013). Overall, exercises bring improvements in cognitive function accompanied by functional and structural changes in brain regions both in normal aging and MCI.

### Electroencephalogram

EEG and ERPs provide higher temporal-resolution biomarkers than neuroimages for cognitive changes. Hogan et al. (2015) measured EEG entropy to uncover the effect of physical fitness on executive function. It was suggested that the effect came from higher functionality of the attentional system in the context of lower levels of frontal EEG entropy. The study repeatedly measured changes in entropy during the 1500 ms post-stimulus interval. ERPs provide closely relationship between cognitive process and electroneurophysiological changes. Regarding the attentional system, Fong et al. (2014) observed P300 amplitude between young adults and older adults with endurance exercises, TCC or sedentary life-style. Compared with older adults with sedentary lifestyle, all other three groups had significantly larger P3 amplitude in task-switching task (Fong et al., 2014). The author concluded that age and participation in physical activity influenced the relationship between physical activity and task-switching (Fong et al., 2014). Attentional network improvement was also shown in aerobic cycling exercise. It resulted in a larger P3 amplitude in the alerting and executive control subtasks across frontal, central and parietal midline sites (Chang et al., 2015). In a psychomotor vigilance task, behavioral and electrophysiological ERPs were obtained and analyzed as a function of time-on-task. Higher-fit participants maintained larger P3 amplitude throughout the task compared to lower-fit who showed a reduction in the P3 magnitude over time (Luque-Casado et al., 2015). Learning is a crucial process in cognitive ability. Long-term potentiation (LTP) represented network plasticity, and worked as an enhancer for learning. In one study, high-activity group maintained amplitude of the N1b after a 30-min rest period, while low-activity group returned to baseline (Smallwood et al., 2015).

Not only one cognitive domain was affected by PE. The amplitude and latency of the visual P1 component and P3a ERPs component evoked in the Oddyball paradigm differed in low and high-intensity exercise group. It was suggested that exercise modulated multiple stages of neural information processing, ranging from early stage sensory processing (P1) to post-perceptual target categorization (P3a) (Bullock et al., 2015). In a retrospective study for aging and physical activity, 50-years lifelong physical activity was associated with faster recall of stimulus-response sets (P2), enhanced response selection during interference processing (N2) and working memory updating (P3b) leading to lower mixing and switch costs (Gajewski and Falkenstein, 2015).

However, cognitive impairment was also observed during both low and moderate-intensity exercise for the flanker task trials that require greater cognitive control. Interestingly, ERPs revealed increased N2 and P3 amplitudes during both exercise conditions relative to rest. The author suggested divergent effects of exercise on behavioral performance measures, accompanied by an upregulation of cognitive control during aerobic exercises (Olson et al., 2015). The study only evaluated the effect of 3-day exercise in healthy participants, regardless of long-term effect.

### Magnetoencephalography

Functional connectivity can also be determined from MEG, which is a much more direct way to measure neural activity. The brain network is a ‘smallworld,’ which combined local segregation with global integration (Watts and Strogatz, 1998; Sporns and Zwi, 2004; Stam, 2004; Bassett et al., 2006). Brain network topology is disturbed in aging and MCI (Bullmore and Sporns, 2009; Stam and van Straaten, 2012). Increased physical fitness was related to better functional brain network topology. Douw et al. (2014) proved that physical fitness was related to modular network topology based on MEG in healthy subjects. The increased intermodular connectivity was associated with better cardio respiratory fitness and better mental fitness, while having less within-module connections. Thus, MEG also showed exercise-related improvements in brain network functions.

### Positron Emission Tomography

Hypometabolism in the DMN is an important [^18^F]FDG-PET marker for the progression of MCI to dementia and has been considered the “metabolic property” of AD (Minoshima et al., 1997; Drzezga et al., 2003; Jack et al., 2013; Fjell et al., 2014). In a study of MCI persons, authors evaluated the effects of a 24-week PE on cognition and rBGM using [^18^F]FDG-PET. Brain metabolic analysis found a bilateral decrease in the rBGM of the dACC, which was negatively correlated with improvements in a visuospatial function/attentional task (Porto et al., 2015). In addition, a study of aging at risk of AD by PiB-PET imaging found that higher cardiorespiratory fitness was related to better cognition, with increased PiB-PET binding and reduced Aβ in CSF, indicating lower risk of developing into dementia (Schultz et al., 2015). Overall, PE improves cognition and changes metabolic networks in areas related to cognition in subjects at risk of dementia.

## Potential Targets for Interventions of Cognitive Decline

From our view of imaging studies on aging and MCI-related changes in brain networks, and exercise-reduced alterations in brain networks, we identify three important networks that might be potential targets for intervention of cognitive decline. The three networks are DMN, FPN and FEN.

### Default Mode Network

The DMN is composed of the posterior cingulate, ventral and superior frontal medial cortices, and bilateral lateral occipital, middle frontal, hippocampal and parahippocampal, and middle temporal cortices (Fox et al., 2005; Buckner et al., 2008). The DMN is supposed to have an important functional role in memory consolidation, self-referential thought, mind-wandering, autobiographical memory (Buckner et al., 2008; Schilbach et al., 2008), and executive control. Increased DMN function has been related to better working memory in young adults (Hampson et al., 2006), and better executive function in older adults (Andrews-Hanna et al., 2007; Persson et al., 2007; Damoiseaux et al., 2008; Voss et al., 2010a), indicating that DMN is an important network for understanding age-related changes in cognition. In addition, DMN is the most relevant network model reflecting the functional and pathological cascade underlying AD (Buckner et al., 2009). The connectivities of the DMN in MCI and AD have been found inefficient (Zhou et al., 2015). However, previous studies showed that exercise could enhance the connectivity of the DMN. Higher connectivity within the DMN had been related to increased cardiorespiratory fitness and exercise intervention increased the DMN connectivity (Voss et al., 2010a,b). Thus, DMN network is a crucial target for interventions of aging and MCI. In order to evaluate the effectiveness and efficiency of PE program, we must pay more attention to the function of the DMN.

### Fronto-Parietal Network and Fronto-Executive Network

Special attention should also be paid to the FPN and the FEN. The inferior parietal cortices, the supplementary motor and primary cortices, the frontal eye-fields, primary and extrastriate visual cortices, the inferior frontal cortex are included in the FPN (Corbetta and Shulman, 2002; Dosenbach et al., 2006). Age-related structural and functional disruptions of the FPN have been found in some studies (Andrews-Hanna et al., 2007; Madden et al., 2007) and could be remediated by PE. The FPN has some overlapping portions with the FEN at the tempo-parietal junction. The FEN is associated with sustained task-set maintenance, error feedback for tuning top–down control, and maintaining action-outcome associations (Rushworth et al., 2004; Dosenbach et al., 2006). It is composed of the anterior prefrontal cortex, insular and frontal operculum cortices, the tempoparietal junction, and the dorsal posterior and anterior cingulate gyri (Dosenbach et al., 2006). Age-related cognitive decline in learning tasks have been found related to the dysfunction of this network (Park and Reuter-Lorenz, 2009), while exercise could alleviate cognitive performance and brain network functionality. Thus, FPN and FEN are important index for the efficiency evaluation of interventions of cognitive decline.

## Future Directions

In the future, longitudinal brain imaging data and the combination of powerful network computational algorithms may generate a new class of progression biomarkers for preclinical dementia. In addition to generating important translational data regarding the systems-level changes that underlie preclinical disease progression, exercise-related changes in functional brain networks may prove efficient and effective PE programs for interventions of cognitive decline. Based on our review, the DMN, FPN and FEN are probably the three most valuable targets for efficiency evaluation of cognitive decline interventions.

## Author Contributions

S-DC designed the whole study and gave suggestions on revising the article. PH and RF searched and selected the studies, analyzed the data, drafted and revised the article. B-YL did some part of writing in EEG data and revised the article. All authors read and approved the final manuscript.

## Conflict of Interest Statement

The authors declare that the research was conducted in the absence of any commercial or financial relationships that could be construed as a potential conflict of interest.

## ABBREVIATIONS

AD: Alzheimer’s disease
Aβ: amyloid-β
CSF: cerebrospinal fluid
EEG: electroencephalogram
dACC: dorsal anterior cingulate cortex
DLPFC: dorsolateral prefrontal cortex
DMN: default mode network
ERPs: event-related potentials
fMRI: functional magnetic resonance imaging
FEN: fronto-executive network
FPN: fronto-parietal network
MCI: mild cognitive impairment
MEG: magnetoencephalography
p-tau: phosphorylated tau
PE: physical exercise
t-tau: total tau
PET: positron emission tomography
